# Therapeutic Role of Neutralizing Antibody for the Treatment against SARS-CoV-2 and Its Emerging Variants: A Clinical and Pre-Clinical Perspective

**DOI:** 10.3390/vaccines10101612

**Published:** 2022-09-26

**Authors:** Manojit Bhattacharya, Srijan Chatterjee, Bidyut Mallik, Ashish Ranjan Sharma, Chiranjib Chakraborty

**Affiliations:** 1 Department of Zoology, Fakir Mohan University, Balasore 756020, India; 2 Department of Biotechnology, School of Life Science and Biotechnology, Adamas University, Kolkata 700126, India; 3 Department of Applied Science, Galgotias College of Engineering and Technology, Knowledge Park-II, Greater Noida 201306, India; 4 Institute for Skeletal Aging & Orthopaedic Surgery, Hallym University-Chuncheon Sacred Heart Hospital, Chuncheon-si 24252, Gangwon-do, Korea

**Keywords:** neutralizing antibody, SARS-CoV-2, pre-clinical trials, clinical trials

## Abstract

Since early 2020, the entire world has been facing a disastrous outbreak of the SARS-CoV-2 virus, with massive reporting of death and infections per day. Medical practitioners adopted certain measures such as convalescent plasma therapy, antibody treatment, and injecting vaccines to eradicate the pandemic. In this review, we have primarily focused on the neutralizing antibodies presently under pre-clinical and clinical trials, focusing on their structures, binding affinity, mechanism of neutralization, and advantages over other therapeutics. We have also enlisted all the nAbs against SARS-CoV-2 and its emerging variants in different phases of clinical trials (phase-1, phase-II, and phase-III). The efficacy of administering antibody cocktails over the normal antibodies and their efficacy for the mutant variants of the SARS-CoV-2 virus in minimizing viral virulence is discussed. The potent neutralizing antibodies have eliminated many of the common problems posed by several other therapeutics. A common mechanism of the antibodies and their relevant sources have also been listed in this review.

## 1. Introduction

The current pandemic of Coronavirus disease (COVID-19) started in the Wuhan province of China in December 2019. During infection, the SARS-CoV-2 virus triggers various immune cascades. Effective, balanced immune components are required to control the pathogenesis of COVID-19 [1,2,3]. The neutralizing antibodies (nAb’s) have shown defense against infected or vaccinated individuals, and can be used as a promising therapeutics component in humans [4]. The nAb is a class of antibodies that neutralize the invading cells of the disease-causing pathogens, thus providing immunity. Such antibodies might be triggered by the use of vaccines or an earlier infection, which are retained inside the body for a longer time than the therapeutic ones. Therefore, neutralizing antibodies are employed for treating several critical pathogenic infections due to their enhanced specificity [5,6].

**Figure 1:**
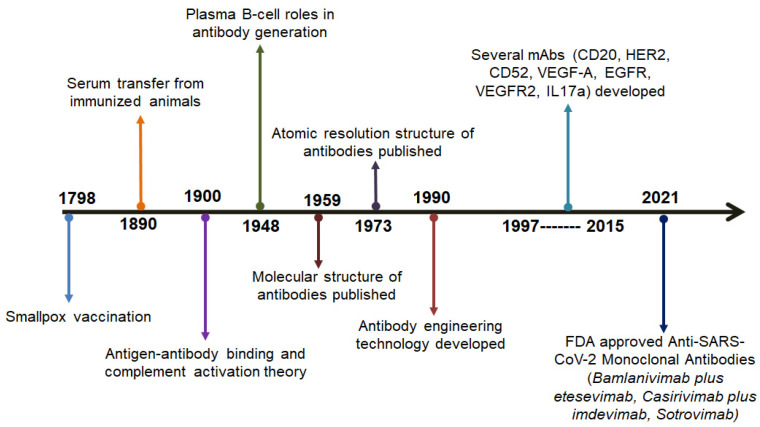
Timelines show different breakthroughs in antibody development.

Before or after viral infection, nAbs can be transferred passively to patients to treat COVID-19 [6]. It has also been proven to be very effective for patients with clinically mild symptoms in the early onset of disease [7]. One of these nAb’s sources is the B cell, isolated from the convalescent plasma donors. An elucidative screening of these antibodies has shown that they can hinder viral entry and prevent SARS-CoV-2 infection [8,9,10]. Some of these nAbs can be derived from humanized mice or convalescent patients and follow the same action mechanism [11]. Antibody development events from the smallpox vaccination were a breakthrough, and this event has shown a new direction for the treatment of COVID-19 as an anti-SARS-CoV-2 mAb (Figure 1). Events that led to the development of antibodies are shown in Table 1. nABs have an effective therapeutic role in preventing SARS-CoV-2 infection. Considering the antigenic part of the spike (S)-protein, the nAbs are developed, which can be specifically bound to the RBD of S-glycoprotein [12,13,14]. The nAbs development depends not only on the structure but also on the alteration of the protein conformation. The nAb, with accurate structure and conformation, invades the host cells, and it is important for the functionality of the nAbs against the infection of the virus [6,15].

**Figure 2:**
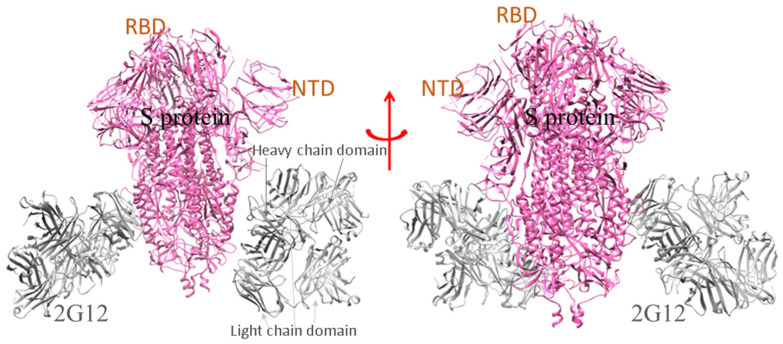
The ribbon model shows the SARS-CoV-2 2P S-protein ectodomain bound to two copies of domain-swapped antibody 2G12 [PDB id: 7L06]. The heavy chain domain of Ab(2G12) interacts with the NTD part of the S-protein.

**Figure 3:**
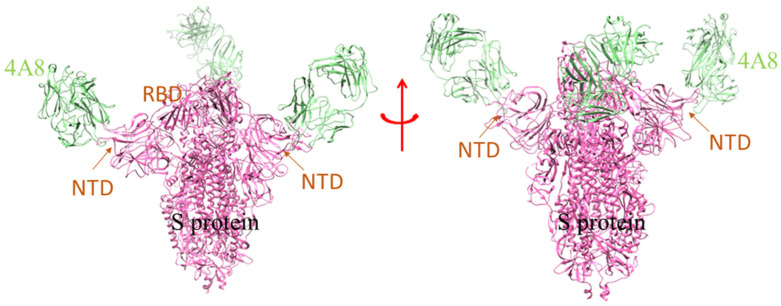
The model demonstrates the binding of human mAb (4A8) to the NTD of S-protein of SARS-CoV-2 [PDB id: 7C2L]. The chains of the mAb unit interact with the different NTD of the S-protein trimeric sub-unit domain.

**Figure 4:**
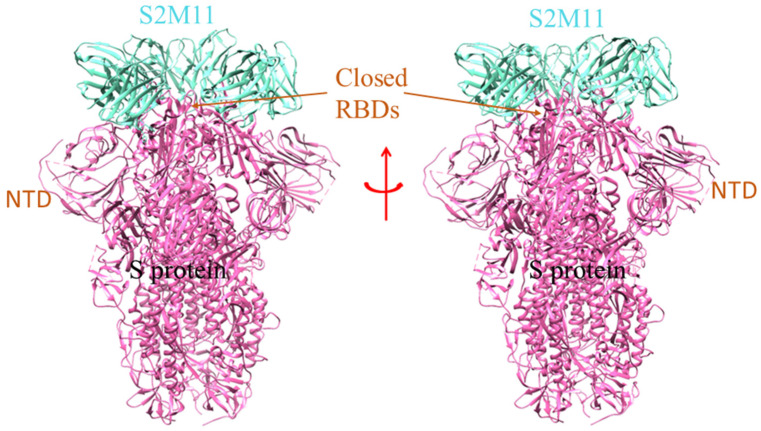
The model shows the human nAb (S2M11) binding with the adjacent part of RBD in closed conformation of S-protein [PDB id: 7K43]. The different domain of SARS-CoV-2 S-protein is marked, where the RBD region interacts with the single unit of nAb (S2M11).

**Figure 5:**
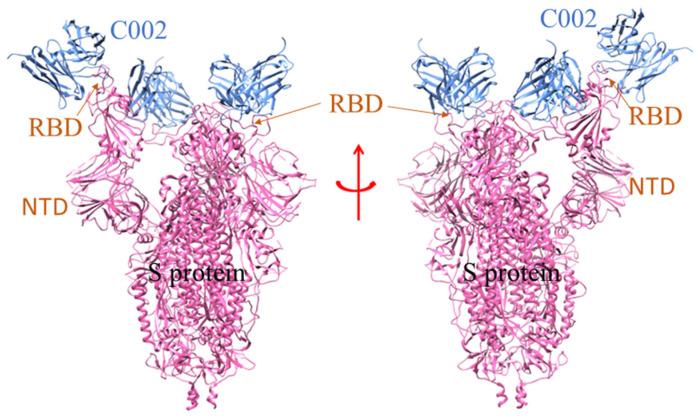
The structure shows the SARS-CoV-2 S 6P trimer in a complex with human nAb (C002) Fab fragment [PDB id: 7K8T]. The nAb (C002) Fab fragment partially interacts with the RBD and NTD regions of the S-protein of SARS-CoV-2.

For the SARS-CoV-2 infection, a few nAbs are highly specific to the S-glycoprotein and prevent the binding of S-glycoprotein to the RBD-ACE2 complex in the host cell. A group of scientists isolated the B cells from the infected individuals and initiated the preparation of different types of nAbs, currently in the pre-clinical and clinical phases (P2C-1F11, BD-368-2, P2B-2F6, COV2-2196, COV2-2130, etc.) [6,26,27]. A model shows where the SARS-CoV-2 spike glycoprotein (S-protein) ectodomain is bound to two copies of domain-swapped natural antibody 2G12 (Figure 2). Likewise, SARS-CoV-2 S-protein in trimeric form also makes a complex with the human nAb (C002) Fab fragment (Figure 3). Many of the nAb isolated from human beings had proven to be effective in treating the SARS-CoV-2 infection in several animals, namely the transgenic mice, hamsters, etc. The nAb (S2M11) Fab part bind with the adjacent receptor-binding domains of S-glycoprotein present in a closed conformation (Figure 4). Another nAbs, named Vh–Fc ab8, targets the spike RBD, which is also very effective in treating the infection [17,18,19]. However, researchers developed some nAbs with a different target site other than the spike RBD. They also targeted other regions besides RBD in the S-protein, and are currently entering the pre-clinical stages [6,28]. It has been noted that nAbs targeting the spike RBD are more efficient than the nAb targeting the other regions of S-protein (other than RBD). At the same time, scientists have shown, through electron microscopy, that the nAb can also bind to the NTD of S-protein. One such example is mAb (4A8), which binds with the S-protein NTD part (Figure 5). Therefore, the NTD might be a potent target for therapeutic mAbs against the COVID-19. This target specificity of the spike RBD has not only proved to be effective for the wild-type strain but also for several emerging mutant variants. They have very minimal immune escape property [13,29]. Liu et al. stated that RBD is a highly conserved region, and researchers should develop more nAb targeting RBD to treat the infection [30].

It has been noted that various nAbs were employed to treat SARS-CoV infection previously, and these nAbs are also used for neutralizing the SARS-CoV-2 infection. However, some of the nAbs of the SARS-CoV virus fail to target the spike RBD region of the SARS-CoV-2 and are unable to neutralize the future viral infection [6]. Some mutational modifications in the SARS-CoV-2 variants may affect the viral infectivity, and also, the mutation-related S-protein configuration change might alter the nAbs binding affinity. On the other hand, it was also recorded that the protein modifications can make them efficient in targeting the conserved epitopes of spike RBD which could enhance the neutralizing capacity of nAbs [31,32,33]. The researcher also reported that the nAbs inhibit the interaction of spike protein with the ACE2 receptor preventing membrane fusion [34]. It is also noted that some of the RBD targeting nAb or the non-RBD targeting ones are incompatible with preventing spike protein interaction against the ACE2 receptor. These nAbs exhibit the viral neutralizing capacity and bind to other S-glycoprotein regions, preventing the entry of the SARS-CoV-2 virus [35]. Several scientists analyzed the detailed phenomena of incapability of nAb to prevent viral entry. They found that these nAbs interact with the Fcγ receptor and lead to the antibody-dependent enhancement (ADE) with the target cells. This ADE formation subsequently leads to the release of cytokines such as IL-6 [36]. However, there are no reports of ADE formation in patients with SARS-CoV-2 infection till today [6].

Given the therapeutic potential of nAbs in viral protection, here, we summarize the nAbs presently under pre-clinical and clinical trials for COVID-19 treatment with special attention to their structures, binding affinity, mechanism of neutralization, and advantages over other therapeutics. Subsequently, we also highlighted the efficacy of administering antibody cocktails over the normal ones and their efficacy against the significant mutant variants of the SARS-CoV-2 virus. The specific role of potent single domains antibodies is also discussed for therapeutics. A collective mechanism of the neutralizing antibodies and their sources are also listed in this review.

## 2. Structure of a Neutralizing Antibody

One of the common features of all the nAbs is the CDRH3 region (complementarity-determining region 3) in the heavy chain. The CDRH3 region comprises of a few gene segments with unique amino acid residues. The three genes present are V (variable), D (diversity), and J (joining). Several studies highlighted that the antibodies interact with the antigens and elicit the immune response, which solely depends on the CDRH3 region [37]. The researcher also studied the CDRH3 region of the antibodies released from the B cells elicited by the spike glycoprotein of SARS-CoV-2. They did not find any significant variation in the length of the CDRH3 region in antibodies compared to the normal population. However, they found that the average length of these isolated CDRH3 regions was nearly 20 amino acids long [37].

**Figure 6:**
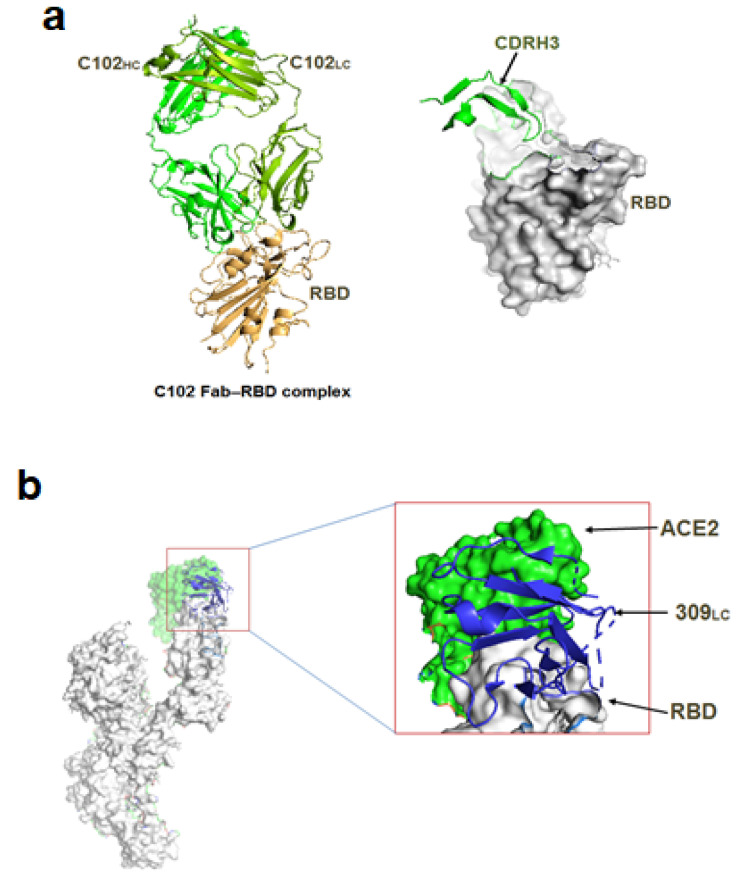

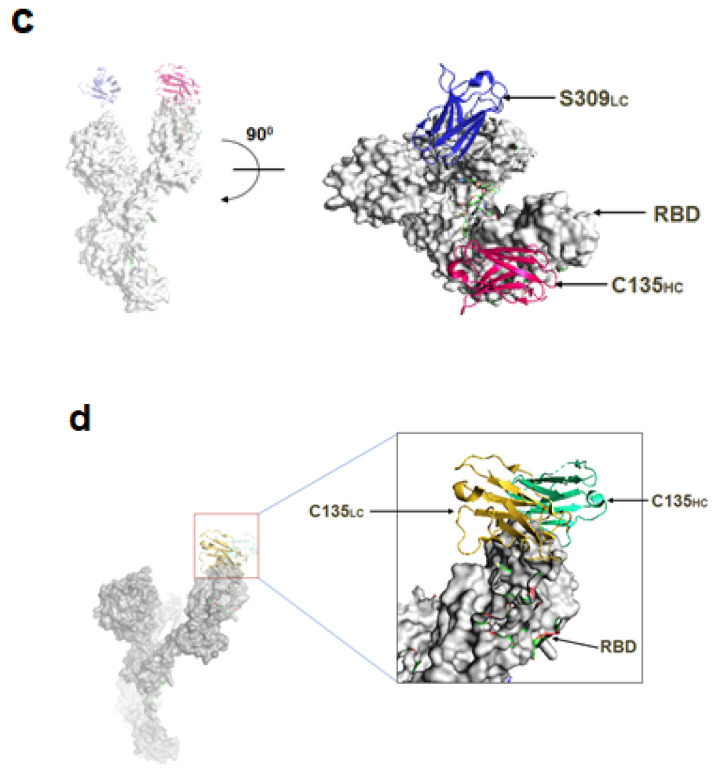
Different types of nAb based on structural and functional attributes. (**a**) Class I nAb (CDRH3) binds with open RBD (C102 Fab-RBD complex). (**b**) Class II nAb S309 bind with both ACE2 and RBD (309LC-ACE2 RBD complex). (**c**) Class III nAb C135 bind distinct RBD part (C135HC and S309LC with RBD). (**d**) Class IV nAb C135 bind with only up conformation of RBD (C135_HC_ and C135_LC_ with RBD). [PDB id: 6WPS, 7K8M].

The nAbs isolated from the convalescent plasma donors are specific to the RBD epitopes. However, some of these nAbs might target overlapping epitopes [38]. These antibodies protect the virus from interacting with the ACE2 receptor preventing viral infection. Like other antibodies, the nAbs employed specifically for preventing the SARS-CoV-2 infection also comprise two chains: heavy and light. The heavy chain is segmented into smaller regions which are encoded by the VH3-53 or VH3-66 genes. In addition, it comprises three complementary determining regions of the heavy chain (CDRH), namely CDRH1, CDRH2, and CDRH3. The CDRH3 region is generally shorter in length than the other two regions [37]. One in vitro study isolated the SARS-CoV-2 nAb and found that it possesses a similar target and comprises VH3-30 genes in the CDRH3 region [37]. According to the structural and functional attributes, Barnes et al. have categorized the nAbs into four types which are:

i. VH3-53 encoded gene that blocks the host ACE2 only in the ‘up’ conformation of the RBD. They exhibit a shorter CDRH3 region (Figure 6a).

ii. Another class of ACE2 blocking antibody is functional in both (up and down) RBD conformations and can even contact adjacent RBDs (Figure 6b).

iii. An additional class of nAb that hinders viral entry by occupying the outer surface of the ACE2; functional in both the up and down conformation of the RBD (Figure 6c).

iv. The fourth class does not interact with the ACE2 receptor and is functional in the ‘up’ conformation of the RBD (Figure 6d) [37].

## 3. Types of mAbs

Remarkably, the beneficial antibodies being cloned in a laboratory need not come from humans but can be from definite animals also. Accordingly, mAbs are of four extensive types.

i. Murine: made from mouse proteins; names of drugs based on this end in -omab.

ii. Chimeric: a combination of mouse and human proteins; names of drugs based on this end in -ximab.

iii. Humanized: here, small doses of mouse proteins are attached to human proteins; names of drugs based on this end in -zumab.

iv. Human: these are fully human proteins; names of drugs based on this end in -umab.

### 3.1. mAb in Treatment of COVID-19

The mAbs have been used to combat MERS, SARS, and other important infections caused by the corona family of viruses in the last decade. Since the COVID-19 pandemic broke out in early 2020, there has been an accelerated drive to use mAbs to fight the virus. The FDA performed a significant role in approving and regulating the use of mAbs to treat COVID-19. Moreover, it was in charge of the guidelines on which mAb-based drugs should be used.

Therefore, in the last two years, a number of drugs are already in use, such as Bamlanivimab and etesevimab, which are used when there is mild to moderate infection by SARS-CoV-2 infection. Casirivimab and imdevimab are used when there is mild to moderate infection and the patient is at risk of developing a severe infection, but the person does not need oxygen therapy. In some previously infected people with the SARS-CoV-2 virus, the immune system goes into overdrive, releasing bursts of proteins known as cytokine storms. While these may or may not fight this virus effectively, they definitely cause severe inflammation in the body, which can be life-threatening. In such a scenario, the antigens from these cytokines must also be suppressed. This is undertaken by a definite category of mAbs called anti-interleukin-6 receptor mABs. Levilimab and Tocilizumab (which are widely present) are examples of such drugs.

Anti-CD6 mAbs are similar to anti-interleukin-6 receptor mAbs, but the biochemical mechanism is slightly different. Itolizumab is an example of such a drug type.

### 3.2. Mechanism of Action of SARS-CoV-2 nAb

**Figure 7:**
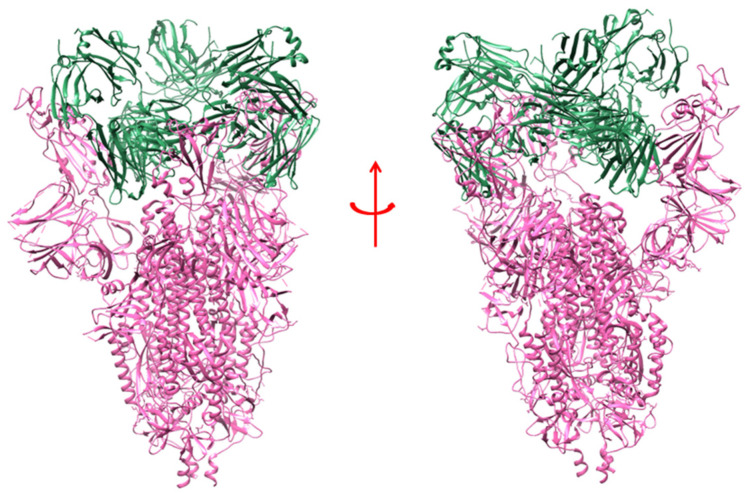
The SARS-CoV-2 S-protein in a complex with a nAb EY6A Fab [PDB id: 6ZDH].

The main target of most of the SARS-CoV-2 nAb is the spike glycoprotein which is responsible for triggering a strong host immune response due to its high antigenicity [38,39,40]. Among the reported nAbs, more than 90% can bind with the RBD and block the viral interaction with the host ACE2. According to Jin D et al., the binding of the nAb to the ACE2 receptor can be subdivided into two regions, namely a/b. The binding affinity of the nAbs to these two sites is expected to inhibit a potent neutralizing effect in hindering the binding of the spike protein. Moreover, these antibodies are unable to bind with the RBD in the same manner. The variable binding of these antibodies helps segregate them [41]. Therapeutic monoclonal antibodies can interact with one or more epitopes of the RBD region [42]. This variation of neutralization in the case of nAb also varies with respect to diverse interaction sites of antigens. For instance, the mAb named S2H13 possesses neutralizing activity by identifying the conformation of the spike protein. However, it has been noted that the EY6A and ACE2 neutralization have different action mechanisms. It has been revealed that during neutralization, EY6A binds to the lower portions of the b region of ACE2 and thus is unable to interact with S1 and S2 junctions of S-glycoprotein. Therefore, these event makes the epitope incapable of binding with the ACE2 receptor (Figure 7). Usually, the nAbs that target a single epitope alter the conformation of the spike glycoprotein (in a down conformation). It helps to make it inaccessible to interact with the ACE2 receptor [43,44]. One of the major reasons enabling the neutralization of the SARS-CoV-2 virus is a creation of a stearic obstacle due to the orientation of the nAbs. This obstacle makes the spike protein incapable of interacting with the host receptor. S2A4 has a very strong viral neutralizing capacity compared to the other nAb’s. After binding with the spike RBD, this antibody causes the shedding of the S1 subunit, hindering the access to bind with the ACE2 receptor. Some nAbs use three spike epitopes to restrain the interaction with the ACE2 receptor. These antibodies surround the RBD in many ways. They bind to the edges and tip of the RBD and use either the heavy chain or light chain to interact with the viral epitopes [41].

Here, in the earlier section, we have discussed that the nAbs target the RBD and NTD (N-terminal domain). The exact mechanism of NTD attachment is not vividly elucidated to date. However, the structural analysis of the spike protein showed that these nAbs bind with the NTD and alter the RBD conformation (down conformation). This phenomenon creates a stearic obstacle coinciding with the antibody and its binding to the ACE2 receptor. Consequently, the spike protein is unable to interact with the ACE2 receptor due to nAb-ACE2 complex [31].

## 4. Advantages of nAb over Vaccines and Convalescent Plasma Therapy

During the pandemic, one of the alarming situations prevailing throughout the world is the evolving SARS-CoV-2 variants and their strategy to immune escape. In this regard, the application of nAbs might be more effective than vaccines. The administration of two or more antibodies (antibody cocktails) together has proven too efficient in the case of evolving variants [45]. Moreover, a more efficient approach must be taken to develop vaccines against the evolving variants to eradicate the pandemic.

Researchers aim to establish the COVID-19 treatment using several nAbs by replacing convalescent plasma therapy (CPT). The primary reason behind replacing CPT with nAbs is the elimination of some blood diseases which are generally the side-effects of CPT. The use of the nAbs helps in the faster development of epitope-specific antibodies. Moreover, a proper dosage of these nAbs forms a high titer of antibodies within a very short time compared to CPT. The high efficacy of nAbs has also proven to have superior results in cases of COVID-19 and certain other disease outbreaks [11].

It has been noted that the FDA approved the CPT to treat hospitalized COVID-19 patients; however, more accurate results are awaited from the undergoing clinical trials. Patients undergoing plasma transfusion should not be comorbid and should not have any chance of antibody-dependent enhancement (ADE). In this regard, nAbs are highly effective against the CPT treatment [46]. We have listed different nAbs in different phases of clinical trials (Table 2).

## 5. Different nAbs Employed for Treating SARS-CoV-2 That Are in Clinical Trials

We have listed different nAbs that are currently in clinical trials (Table 2). The detailed account of these nAbs is also discussed in the following sections.

### 5.1. JS016

Etesevimab (also known as JS016) is a neutralizing monoclonal antibody possessing certain replacements in the amino acid residue (L234A, L235A) in the Fc region, which prevents the interaction of the S glycoprotein with the ACE2 receptor. This mAb also aims to prevent host–cell invasion and viral replication. It belongs to the class of IgG1 isotype and the LALA mutation in the Fc region that prevents various properties such as antibody-dependent cellular cytotoxicity and antibody-dependent enhancement. It mitigates the activation of macrophages to diminish the excessive cytokine storm observable in severely affected COVID-19 patients, proving the effectiveness of the antibody. An in vitro study conducted on a macaque model reported satisfactory results of JS016 in preventing SARS-CoV-2 infection [47].

### 5.2. MW33

The MW33 is a nAb of the type IgG1κ, highlighting several essential features for preventing COVID-19 disease. The rhesus monkey was the animal model where this particular antibody was administered. The MW33 antibody targets the spike RBD, but the conventional cytochrome P450 enzymes do not mediate its expulsion from the body. Instead, it is carried out by some non-specific proteolytic enzymes. The Phase I clinical trials of the MW33 have shown some decrement in the biochemical parameters. Later stages of the clinical trials will be performed to conclude more about the MW33 antibody to assess its safety, tolerability, and other important profiles [48].

### 5.3. CT-P59

The CT-P59 is a nAb obtained from patients who had convalescent plasma therapy for treating the SARS-CoV-2 infection. This antibody hinders the interaction of the spike RBD with the host ACE2 receptor, and this antibody inhibits the viral replication capacity, thus, reducing the viral load. The Phase I trial was conducted in two stages to establish the safety, tolerability, and pharmacokinetic profile of the CT-P59 antibody in healthy volunteers as well as patients with mild symptoms. It is also proved to be very efficient against the evolving variants. For example, the administration of CT-P59 proved to reduce the viral load in the respiratory tract (upper and lower) against the Beta variant. Further trials are also in the process that will elucidate more about the safety, tolerability, and pharmacokinetics profiling of the antibody [49].

### 5.4. REGEN-COV

According to the information obtained from the reports of the three clinical trial phases, REGEN-COV proved to be an efficient nAb for COVID-19 disease. The first two phases indicate that the administration of REGEN-COV has potentially lowered the rate of hospitalizations for COVID patients. In addition, it also hinders viral replication capacity, leading to a lower viral load. REGEN-COV was able to lower the rate of mortality as well as intensive care support, and it reduced the symptoms caused by the SARS-CoV-2 infection. The REGEN-COV also proved to be a very efficient mAb for several emerging SARS-CoV-2 variants, namely the Alpha (B.1.1.7), Beta (B.1.351), Gamma (P.1), and Delta (B.1.617.2) variants. In late 2020, due to the increased efficacy, REGEN-COV also achieved emergency approval from the FDA for administration in the SARS-CoV-2-infected patients with mild and moderate symptoms who did not require hospitalization support [50].

### 5.5. LY3819253/LY-CoV555

The administration of LY3819253 nAb was mainly on COVID-19 patients whose symptoms ranged from mild to moderate. The trial report indicated a reduction in the patients’ viral load administered with the antibody compared to the placebo. Moreover, the safety profile analysis for the antibody is very convincing, suggesting it to be an efficient treatment method for COVID patients in an emergency. Even it proved its superiority in the various zones having high-risk patients. The administration also reduced the rate of hospitalization, in turn reducing mortality. In addition, the administration of LY3819253 showed no adverse effects other than diarrhea and vomiting in a few volunteers. It also possesses special features responsible for viral clearance within a very short time [51].

### 5.6. VIR-7831

The VIR-7831 is similar to the S309 antibody isolated from the patients who recovered from SARS-CoV-2 infection. S309 has also shown quite a good result in neutralizing the SARS-CoV-2 virus. VIR-7831 is being modified to enhance its ability to recognize the SARS-CoV-2 virus. The main aim behind engineering VIR-7831 is to make it capable of recruiting cytotoxic T cells, killing the virally infected cells effectively. In addition, antibody engineering will strengthen the safety, tolerability, and pharmacokinetic profile of VIR-7831. However, this antibody administration results in certain adverse outcomes, notably the formation of anti-drug antibodies against VIR-7831. Further trials are still undergoing and are expected to overcome the flaws, and therefore, VIR-7831 will be an effective treatment against treating the pandemic [52].

### 5.7. BGB DXP593

The exact mechanism that enables the BGB-DXP593 antibody to inhibit the SARS-CoV-2 virus entry into the host cell is not completely known. However, by analyzing the structural similarity of the SARS-CoV and SARS-CoV-2 researchers expect that the antibody employed for neutralizing SARS-CoV could be potentially be useful in treating the SARS-CoV-2 infection. This antibody possesses a complementary region named CDR3H, which targets the spike RBD. BGB-DXP593 mainly inhibits viral entry by CDR3H. Currently, the Phase 2 trial of this antibody is elucidating more about its efficiency and safety profile in preventing SARS-CoV-2 infection. This is mainly applied to COVID-19 patients having mild to moderate symptoms [52].

### 5.8. SCTA01

Like Etesevimab, SCTA01 also possesses LALA modification in the Fc region and hinders the interaction between the spike protein and the ACE2 receptor. It is also known as HB27, and is a member of the IgG1 antibody isotype. It shares some common functionality with the Bamlanivimab. The amino acid residue (LALA) mutation is responsible for eliminating the antibody-dependent cellular cytotoxicity and antibody-dependent enhancement properties. The pre-clinical reports of SCTA01 elucidated the safety and antiviral characteristic of this mAb. All the potential effects seen in the volunteers were mild and did not require additional support to cure them. The in vitro study of this nAb on mice and rhesus monkeys also highlighted its ability to lower the viral load [53].

### 5.9. DZIF-10c

The DZIF-10c is one of the most potent antibodies employed for treating the SARS-CoV-2 infection. The evolving mutations in several VOCs and VOIs are proven to impart immune escape properties. Studies have shown that DZIF-10c effectively neutralizes the virus in 16 prevalent mutations. The neutralizing inability was only observed in the case of the K444Q mutation. DZIF-10c possesses a much greater antiviral characteristic than the other antibodies, suggesting a more efficient neutralization. This antibody’s safety and pharmacokinetic profiling highlighted its use for clinical purposes. In addition, the extended half-life and greater loads of neutralization titers make it more suitable for clinical usage. It is completely capable of neutralizing the SARS-CoV-2 infection by the alpha variant (B.1.1.7), and it also plays a pivotal role in neutralizing the infection by the beta variant (B.1.351) [54].

### 5.10. SAB-185

According to the Phase I clinical trial reports, SAB-185 has shown a convincing safety and tolerability profile for future use. It is a very potent, full-human polyclonal antibody capable of neutralizing most of the evolving mutations. The common immune escape property of S477N, D614G, N501Y, and E484K are eliminated by the administration of SAB-185. This antibody is isolated from the specially engineered bovines by hyperimmunization. This polyclonal antibody can recognize a series of epitopes in the spike antigen. As a result, single-point mutations cannot alter their neutralizing capacity. This antibody has provided a potent neutralization for SARS-CoV-2 and many viruses, namely Ebola, Haantan, MERS-CoV, etc. [55].

### 5.11. COR-101

COR-101, also known as STE90-C11, targets the ACE2-RBD complex, affecting viral entry. The neutralizing effect of this antibody notably brings no difference in the case of the mutations in the RBD. These antibodies share some common features with the human germline genes (VH3-66 family). The selection of COR-101 is better for SARS-CoV RBD because it does alter its conformation, and there is no problem of any stearic clash. COR-101 also interacts with the CB6 and B38 epitopes of RBD, such as the other antibodies. COR-101 has successfully treated patients with mild to moderate infection symptoms. Moreover, another advantage of COR-101 is its interaction with the 473 to 476 amino acid residues, a harbor for many evolving mutations. It has shown a greater tolerance for most of the evolving variants such as Kappa, Delta, etc. [56].

### 5.12. Bamlanivimab and Etesevimab

This antibody cocktail comprises two mAbs, Bamlanivimab and Etesevimab resulting in the spike protein binding with the Fc fragment of the two and making it more efficient. A comparison of the effects of these nAbs with the placebo group after 3 to 11 days significantly reduced the viral load, and a minimal amount of people infected with COVID-19 required hospitalization support. A report from the Phase III clinical trial also indicated the efficiency of this antibody cocktail in high-risk groups of people. This nAb cocktail reduced the hospitalization and death rate by up to 70% compared to the placebo group [11].

## 6. nAbs Employed for Treating SARS-CoV-2 and Are in Pre-Clinical Trial

The numbers of nAbs against SARS-CoV-2 infection in the pre-clinical trial are listed in Table 3.

### 6.1. AR-712

The antibody cocktail is a way of treating the present VOCs and VOIs. AR-712 is a cocktail that efficiently neutralizes the dominating Delta variant. This mAb cocktail, developed by Aridis Pharmaceuticals, was self-administered to COVID-19 patients and did not require any hospitalization. This cocktail was identified from the convalescent plasma of the SARS-CoV-2 infected patients. It consists of two IgGs isolated from the B-cells of these patients [57].

### 6.2. IMM-BCP-01

The IMM-BCP-01, developed by Immunome Inc., is an antibody cocktail with great potential in neutralizing the Delta variant of the SARS-CoV-2. According to the reports published in late July 2021, this nAb will enter the first phase of the clinical trial. It is a cocktail functional with three mAbs and targets nearly three non-overlapping epitopes of this virus. It will also collaborate with the US FDA to submit an Investigational New Drug Report before entering the clinical trial [57].

### 6.3. SPKM001

An anti-SARS-CoV-2 nAb developed by SpikImm and Institut Pasteur is the SPKM001. It is expected to enter the clinical trial in Europe, Brazil, and North America by early 2022. It has effectively neutralized most VOCs and VOIs such as Alpha, Delta, Beta, Gamma, and Delta Plus. It has a strong binding affinity towards the RBD of SARS-CoV-2, thus hindering the interaction of these variants with the host receptors [57].

## 7. New Emerging SARS-CoV-2 Variants and Possible Therapeutic Interventions

With the advent of time, several new mutations have accumulated in the SARS-CoV-2 viral genome. These mutations have altered the characteristics of the virus in terms of transmissibility and infectivity. On this basis, the WHO and CDC categorized emerging variants as VOC and the VOI. Several mutations in these variants confer the ability to escape the mAbs and vaccines [58,59].

### 7.1. B.1.1.7 (Alpha)

This variant was first detected in the UK. Several mAbs, including antibody cocktails, have been found to be very potent in neutralizing this variant. For instance, the COVOX-222 has an efficient adequate neutralizing system. It interferes with the amino acid substitution at the 417 positions and neutralizes this variant efficiently [59]. Due to the presence of the N501Y mutation, the interaction with ACE2 and RBD is strengthened, facilitating increased neutralization. Another unique antibody cocktail, LYCoV-555 (Bamlanivimab + Etesevimab), has a very strong binding affinity with the RBD in both possible conformations and is not afflicted by the substitution of Y501. According to Jiejie Geng and his colleagues, CD147 is very active in blocking viral entry into the host cells [60]. CD147 has a neutralization efficiency nearly equal to 69% at a certain concentration. It also prevents the building of cytokine storms in individuals infected with the virus. A combination of casirivimab and imdevimab has shown efficient neutralization efficiency in the case of the Alpha variant. This combination attaches to both sides of the RBD of the S-glycoprotein [60,61].

### 7.2. B.1.351 (Beta)

This lineage isolated from South Africa possesses several missense mutations and deletions. The neutralizing mAb MG1141A has been extremely proficient in neutralizing the B.1.351 variant. In addition to neutralization, MG1141A also plays a pivotal role in viral clearance by utilizing the immune cells’ property to undergo phagocytosis [62]. As previously stated, CD147 functions to prevent the entry of the Beta variant. In the case of B.1.351, the neutralization efficiency is nearly 75%. Another antibody cocktail, Tixagevimab and Cilgavimab, is very potent in dominating the B.1.351 variant [63]. It can significantly identify the non-conserved epitopes residing in the RBD of the S-glycoprotein and inhibit the viral entry into the host cells.

### 7.3. P.1 (Gamma)

The first evidence of the Gamma variant was made in Manaus, Brazil. It consists of several mutations, making it inevitable that the available antibodies will neutralize it. Many antibodies neutralizing the alpha and beta variants have also efficiently blocked P.1 [60]. The mAbs such as COVOX-222 and COVOX-253 follow a common mechanism of neutralization, i.e., they interact with the ACE2 receptor, making it difficult to bind with the RBD of the spike protein. However, casirivimab, a potent nAb, is inefficient at blocking the Gamma variant. It needs to be combined with imdevimab to accelerate the neutralization efficiency [64]. The most common antibody that can neutralize most of the evolving SARS-CoV-2 variants is CD147 or meplazumab. In the case of P.1, its neutralization efficiency stands at nearly 50% [59].

### 7.4. B.1.617.2 (Delta)

This VOC, which dominated the second wave of the COVID-19 pandemic, was isolated from India. It differs from the other VOCs to a greater extent, possessing a single mutation (D614G) common with the others. The exclusive mutations L452R and T478K make this variant extremely contagious with more virulence. According to the data highlighted in Table 4, it is evident that the commonly used antibodies for Alpha and Beta variants have shown significant neutralization efficacy for Delta. The mechanism of action of these neutralizing antibodies is also the same in this case, as discussed earlier.

### 7.5. B.1.1.529 (Omicron)

According to Zeng et al., the Omicron variant confers a wider ability to escape antibodies compared to the other variants [65]. The structural modeling and sequence-based study also stated that the improved binding affinity of Omicron S-protein with the hACE2 receptor caused increased virulence [66]. The Omicron variant has more mutations than any other previously reported SARS-CoV-2 variant. It possesses 50 mutations, out of which 32 pertain to the spike protein, which is the target site for most vaccines to neutralize the virus. Many mutations are novel and not found in the previous viral variants. Specifically, the variant is characterized by 30 amino acid changes, three small deletions, and one small insertion in the spike protein compared with the original virus, of which 15 are located in the receptor-binding domain (residues 319–541) [67]. The nAbs Sotrovimab, Paxlovid, and molnupiravir have shown efficiency in the case of this variant. Due to a large number of residing mutations, the Omicron variant is highly resistant to antibody cocktails [68,69,70]. Many resistant mutations residing in the spike protein of the Omicron variant are responsible for lowering the Ab titers elicited by the vaccination [71]. Rather, a single antibody is more efficient in combating the SARS-CoV-2 infection. Sotrovimab is extremely efficient in binding to the conserved antigenic epitopes rather than the non-overlapping ones. However, the most efficient antibody that can combat the Omicron variant is molnupiravir. Upon entering the host cell, molnupiravir interferes with the viral replication of the B.1.1.529 variant, a unique property that is possessed by an antibody [72]. Thus, molnupiravir can neutralize this variant to a greater extent.

## 8. Heavy Chain Antibodies (HCAbs) against SARS-CoV-2

**Figure 8:**
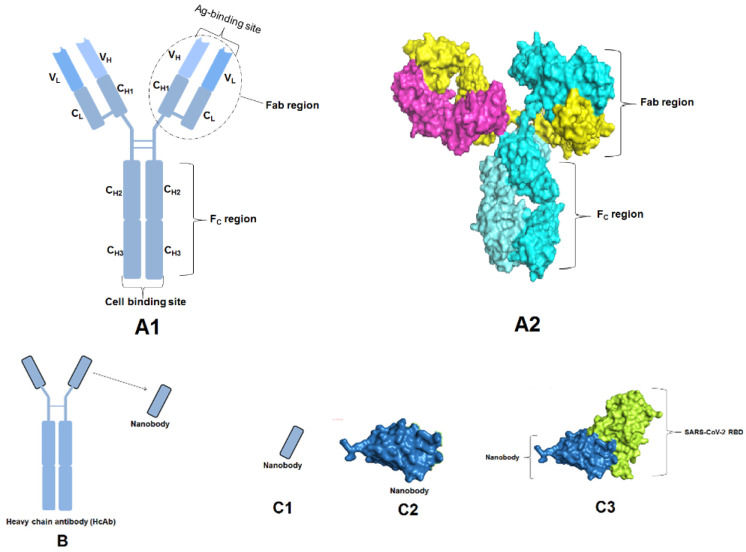
Structure of heavy chain antibodies and its parts. (**A1**) Schematic structure depicted different parts. (**A2**) Surface structure shows Fc and Fab region. (**B**) Heavy chain antibodies along with the nanobody. (**C**) Nanobody complex with the SARS-CoV-2 RBD.

Heavy chain antibodies (HCAbs) are specialized active antibody fragments that are not associated with the light chains, and their VH (variable heavy) regions are also functional as a part of a single unit (Figure 8(A1,A2)) [75]. The VH regions serve as perfect building blocks for several antibody-based treatments as they permit the addition of molecules in sequence to construct multispecific antibodies. The HCAbs possess a unique paratope that interacts with the variable domain of the heavy chain without involving any light chain domains (Figure 8B). These classes of antibodies originated from the camelid species and were found to be extremely proficient in treating COVID-19. They are more immunogenic than conventional ones and possess some unique physical properties that help in the larger production of these antibodies. These HCAbs, however, have a lower affinity for binding with the antigenic epitope and are easily excreted by the kidney. The HCAbs (especially nanobodies) can even be used to detect the presence of the SARS-CoV-2 virus (Figure 8(C1–C3)). These nanobodies are capable of being inhaled by the patients, and, thus, can be used to prevent viral replication in the lungs. Mostly, the RBD of the spike protein is a potential target of HCAbs [76,77].

## 9. Single Domain Antibody against SARS-CoV-2

To establish an efficient therapeutic against the SARS-CoV-2, heavy chain single domain antibodies (sdAb) have shown promising results. Experiments suggest that the competitive binding of these sdAbs plays a pivotal role in hindering the interaction between the hACE2 receptor and the viral RBD [75,78]. Moreover, fusing the IgG1 Fc with these sdAbs accelerates their neutralizing efficiency to a greater extent. These antibodies, also called nanobodies, have the antigen-binding capacity to a greater extent, making them an effective tool for designing therapeutics to eradicate this global outbreak. These sdAbs are cost-effective and comparatively more stable than nAbs [75]. Considering the present scenario of COVID-19 treatment with the administration of certain vaccines and antibody therapy, the development of the sdAbs will be more susceptible to treating the infection. The administration of these antibody cocktails is believed to give a more durable protective response than the current therapeutics [78]. According to several pieces of research, sdAbs are extremely efficient in targeting the epitopes in the RBD of the SARS-CoV-2 variants [75]. These epitopes, in turn, are responsible for the extremities caused to human health upon the viral entry. The extraordinary feature of these antibodies makes them compatible with being used as a particle delivery system. sdAbs can be delivered into the lungs through nasal delivery as well as the gastrointestinal tract to prevent the interaction of the virus with the ACE2 receptor [79,80]. Studies also suggest that IgA is a better neutralizing tool than IgG; thus, the fusion of IgA with the sdAb’s will be extremely efficient in serving as a diagnostic tool to eradicate the pandemic.

## 10. Conclusions

**Figure 9:**
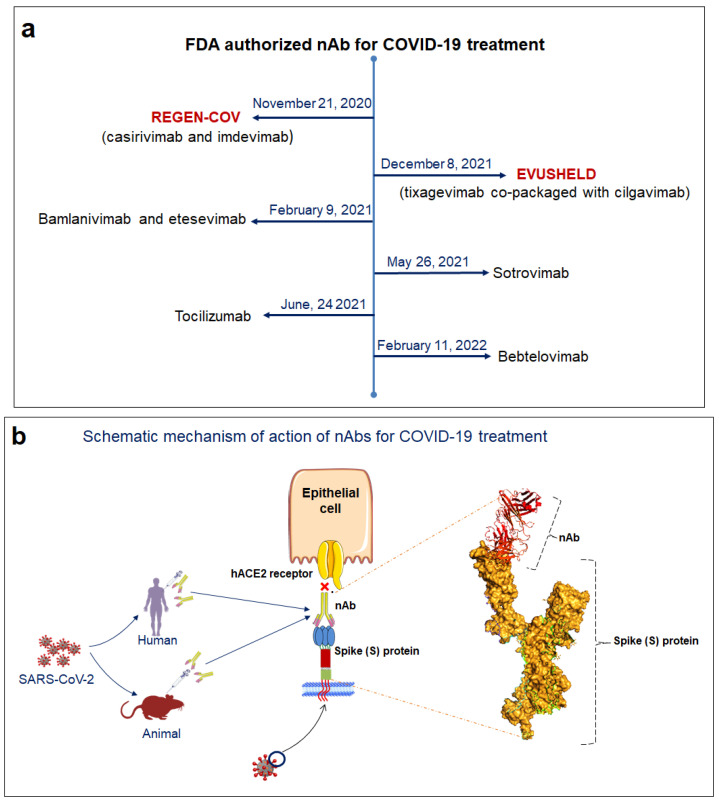
FDA approved mAbs against SARS-CoV-2 and their mode of action. (**a**) Timeline depicting the development of FDA approval for the mAbs against SARS-CoV-2. (**b**) The schematic diagram illustrated the mechanism of action of nAbs. It shows the neutralization of SARS-CoV-2 spike protein.

An efficient strategy to combat this pandemic is the administration of nAbs. The FDA has approved several mAbs for use against SARS-CoV-2. A brief timeline depicting the development of FDA approval for the mAbs against SARS-CoV-2 with their mechanism of neutralization is shown in the Figure 9.

These nAbs have given promising results in minimizing the virulence of the SARS-CoV-2 virus. Several antibody treatments administered by following an appropriate dosage are considered prophylactic measures for treating severe patients. Before the emergence of vaccines, this therapeutic strategy provided a bit of relief to the world in controlling the havoc. Moreover, the administration of combined antibodies (known as the antibody cocktail) has been extraordinarily efficient for the evolving mutants, reducing the chance of escaping the immune system. Several subsets of nAbs isolated from SARS-CoV and MERS-CoV patients had also shown viral neutralization capacity in SARS-CoV-2 patients. Studies also highlight that the administration of these antibodies at an early stage will be more helpful for the population in preventing COVID-19. In turn, high-throughput engineering strategies can be used to construct more neutralizing antibodies with a very high binding affinity, thereby providing great relief for the entire world.

## Figures and Tables

**Table 1:** Events that led to the development of antibodies.

Sl. No.	Year	Scientists Involved	Progress in the Development of Antibodies	Reference
1.	1798	Edward Jenner	The breakthrough of the smallpox vaccine	[16]
2.	1890	Emil von Behring and Shibasabura Kitasato	The transfer of serum to cure diphtheria taken from immunized animal	[17]
3.	1900	Paul Ehrlich	The advancements of several concepts such as antigen-antibody binding, side-chain theory, and complement activation	[18]
4.	1948	Astrid Fagraeus	Elucidated the importance of B cells	[19]
5.	1959	Gerald Edelman and Rodney R. Porter	The publication of the molecular tructures of various antibodies	[20]
6.	1973	D Inbar, J Hochman, and D Givol	The publication of the molecular tructures of the antibodies fragment	[21]
7.	1990	A Plückthun	Antibody engineering	[22]
8.	1997–2015	-	Development of various antibodies such as CD20, HER2, CD52, VEGF-A, EGFR, VEGFR2, and IL17A	[23,24]
9.	2021	-	Development of anti-SARS-CoV-2 antibodies such as Bamlanivimab plus etesevimab, Casirivimab plus imdevimab, and Sotrovimab	[25]

**Table 2:** Different neutralizing antibodies which are in clinical trials.

Sl. No.	nAb	Trial No.	Status	Recruitment	No. of Participants	Sponsor	Country	Allocation	Remarks
1.	JS016	NCT04441918	Phase II	Recruiting	40	Shanghai Junshi Bioscience Co., Ltd.	China	Randomized	A randomized, placebo-controlled study reporting its safety, pharmacokinetics, and immunogenicity administered in healthy subjects.
2.	LY3832479	NCT04441931	Phase II	Completed	26	Eli Lilly and Company	United States	Randomized	A randomized, placebo-controlled study reporting its safety, tolerability, and pharmacokinetics of the mAb in healthy adult volunteers.
3.	LY-CoV016	NCT04427501	Phase II	Active but not recruiting	3290	Eli Lilly and Company	United States	Randomized	A randomized, placebo-controlled study reporting the tolerability, efficiency, and safety profile of the antibody in COVID-19 patients with mild to moderate symptoms.
4.	TY027	NCT04429529	Phase III	Completed	32	Tychan Pte Ltd.	Singapore	Randomized Randomized	A randomized, placebo-controlled, time-lagged study conducted in healthy subjects.
		NCT04649515		Recruiting	1305				A randomised, placebo controlled study of TY027 aimed for treating COVID-19 patients.
5.	BRII-196	NCT04479631	Phase III	Completed	16	Brii Biosciences Limited	China	Randomized	A randomized, placebo-controlled study of BRIL-196 monoclonal antibodies reporting its safety, tolerability, and pharmacokinetics.
6.	BRII-198	NCT04479644	Phase III	Completed	17	Brii Biosciences Limited	China	Randomized	A randomized, placebo-controlled study of BRIL-196 monoclonal antibodies reporting its safety, tolerability, and pharmacokinetics.
7.	CT-P63	NCT05017168	Phase I pending	Not yet recruiting	24	Celltrion	Poland	Randomized	A randomized, placebo-controlled study reporting the tolerability, efficiency, and safety profile of the antibody in COVID-19 patients with mild to moderate symptoms.
8.	XVR011	NCT04884295	Phase I	Recruiting	279	ExeVir Bio BV	Belgium and Italy	Randomized	A randomized, placebo-controlled study reporting the tolerability, efficiency, and safety profile of the antibody in COVID-19 patients with mild to moderate symptoms.
9.	ABBV-47D11	NCT04644120	Phase I	Completed	25	AbbVie	United States	Randomized	A randomized, placebo-controlled study of ABBV-47D11 and ABBV-2B04 monoclonal antibodies reporting its safety, pharmacodynamics, and pharmacokinetics.
10.	HFB30132A	NCT04590430	Phase I	Active but not recruiting	24	HiFiBiO Therapeutics	United States	Randomized	A randomized, placebo-controlled study reporting its safety, tolerability, and pharmacokinetics of the mAb in healthy adult volunteers.
11.	ADM03820	NCT04592549	Phase I	Recruiting	40	Ology Bioservices	United States	Randomized	A randomized, placebo-controlled study reporting its safety, pharmacokinetics, and immunogenicity.
12.	DXP604	NCT04669262	Phase I	Completed	25	BeiGene	Australia	Randomized	A randomized, placebo-controlled study reporting its safety, pharmacokinetics, and immunogenicity in healthy volunteers.
13.	HLX70	NCT04561076	Phase I	Not yet	24	Hengenix Biotech Inc	United States	Randomized	A randomized, placebo-controlled study reporting its safety and pharmacokinetics.
14.	COR-101	NCT04674566	Phase I and Phase II	Recruiting	45	Corat Therapeutics Gmbh	Germany	Randomized	A randomized, placebo-controlled study reporting its safety, tolerability, and pharmacokinetics and immunogenicity of COR-101 in hospitalized COVID patients.
15.	VIR-7832	NCT04746183	Phase I and Phase II	Recruiting	600	University of Liverpool	United Kingdom	Randomized	A randomized, placebo-controlled trial aimed to evaluate the efficacy of the drug in treating COVID-19 patients.
16.	LY-CoV1404, LY3853113	NCT04634409	Phase II	Active but not recruiting	1782	Eli Lilly and Company	United States	Randomized	A randomized, placebo-controlled study reporting the tolerability, efficiency, and safety profile of the antibody in COVID-19 patients with mild to moderate symptoms.
17.	COVI-AMG (STI-2020)	NCT04734860	Phase II	Recruiting	500	Sorrento Therapeutics, Inc.	United States	Randomized	A randomized, placebo-controlled study aimed to evaluate the safety and efficacy of the nAb in patients having mild COVID-19 symptoms.
18.	DXP593	NCT04532294	Phase II	Completed	18	BeiGene	Australia	Randomized	A randomized, placebo-controlled study reporting its safety, pharmacokinetics, and immunogenicity in healthy volunteers.
		NCT04551898			181		United States		A randomized, placebo-controlled study highlighting the neutralizing efficiency of the BGBDXP593 mAb in COVID-19 patients having mild and moderate symptoms.
19.	MW33	NCT04533048	Phase II	Completed	42	Mabwell (Shanghai) Bioscience Co., Ltd.	China	Randomized	A clinical study to evaluate the safety, pharmacokinetics, and immunogenicity of MW33 in normal, healthy volunteers.
		NCT04627584		Recruiting	150				A randomized, placebo-controlled study reporting the efficiency and safety profile of the antibody in COVID-19 patients with mild to moderate symptoms.
20.	MAD0004J08	NCT04932850	Phase II and Phase III	Active but not recruiting	30	Toscana Life Sciences Sviluppo s.r.l.	Italy	Randomized	A randomized study to evaluate the safety, pharmacokinetics, and immunogenicity of MAD0004J08 in normal, healthy volunteers.
		NCT04952805		Recruiting	800				A randomized, placebo-controlled study aimed to evaluate the safety and efficacy profile of the antibody in adult COVID-19 volunteers who were asymptomatic, or had moderately severe symptoms.
21.	C144-LS and C-135-LS	NCT04700163	Phase II	Active but not recruiting	23	Rockefeller University	United States	Randomized	A randomized study to evaluate the safety, pharmacokinetics of two antibodies in normal, healthy volunteers.
22.	SCTA01	NCT04483375	Phase II and Phase III	Completed	33	Sinocelltech Ltd.	China	Randomized	A randomized, placebo-controlled study reporting its safety, tolerability, and pharmacokinetics of SCTA01 in healthy adult volunteers.
		NCT04644185		Recruiting	795		United States		A randomized, placebo-controlled study employed for examining the efficiency of SCTA01 in COVID-19 affected subjects having severe symptoms.
23.	ADG20	NCT04805671	Phase II and Phase III	Recruiting	1084	Adagio Therapeutics, Inc.	Germany, Greece, Brazil, Argentina, Poland, Ukraine	Randomized	A randomized, placebo-controlled study reporting the efficacy of the ADG20 mAb in healthcare workers having mild to moderate symptoms.
		NCT04859517			6412		United States		A randomized, placebo controlled trial for evaluating the safety profile of the antibody in preventing SARS-CoV-2 infection.
24.	AZD7442 (AZD8895 + AZD1061)	NCT04507256	Phase III	Active but not recruiting	60	AstraZeneca	United Kingdom	Randomized	A randomized, placebo-controlled study reporting its safety, tolerability, and pharmacokinetics of AZD7442 in healthy adult volunteers.
		NCT04625725			5197		United States		A randomized, placebo-controlled study employed for evaluating the efficiency of AZD7442 in subjects who are not yet encountered by the SARS-CoV-2 virus.
		NCT04625972			1121		United States		A randomized, placebo-controlled study employed for evaluating the efficiency of AZD7442 in subjects who are already infected by the SARS-CoV-2 virus.
25.	CT-P59	NCT04525079	EUA	Recruiting	32	Celltrion	Republic of Korea	Randomized	A randomized, placebo-controlled study reporting its safety and pharmacokinetics of CT-P59 in healthy volunteers.
		NCT04593641		Active but not recruiting	18				A randomized, Double-placebo-controlled, study reporting the viral nature, safety, and tolerability of the nAb in patients having mild symptoms.
		NCT04602000		Recruiting	1020				A randomized, placebo-controlled study employed for examining the efficiency of CT-P59 in COVID-19 affected subjects having severe symptoms.
26.	VIR-7831	NCT04545060	EUA	Completed	1057	Vir Biotechnology, Inc.	United States	Randomized	A randomized, placebo-controlled study employed for evaluating the safety and efficiency of the nAb for treating COVID-19 patients who did not require any hospital support.
27.	REGN-COV2	NCT04425629	EUA	Recruiting	6420	Regeneron Pharmaceuticals	United States	Randomized	A master protocol study reporting the safety and efficacy of the anti-spike mAbs in healthcare works affected with SARS-CoV-2 virus.
		NCT04426695		Completed	2252				A master protocol study reporting the safety and efficacy of the anti-spike mAbs in COVID-19 positive patients who required hospital support.
		NCT04452318		Active but not recruiting	3750				A randomized, placebo-controlled study evaluating the efficacy and the safety of the mAb in the household contacts to prevent SARS-CoV-2 infection.
28.	LY-CoV555 (LY3819253); combination of LY-CoV555 with LY-CoV016 (LY3832479)	NCT04411628	Phase I	Completed	24	Eli Lilly and Company	United States	Randomized	A randomized, placebo-controlled study reporting its safety, tolerability, and pharmacokinetics of LY3819253 in hospitalized COVID-19 patients.
		NCT04427501	Phase II	Recruiting	3290				A randomized, placebo-controlled study highlighting the neutralizing efficiency of the mAb in COVID-19 patients having mild and moderate symptoms.
		NCT04497987	Phase III	Completed	1374				A randomized, placebo-controlled trial highlighting the safety and efficacy of the mAb alone and in combination to evaluate the immune response in nursing staffs to prevent SARS-CoV-2 infection.
		NCT04501978	Phase III	Recruiting	10,000	University of Minnesota			A randomized, blinded controlled trial reporting the safety and efficacy of the COVID-19 positive patients who required hospital support.
		NCT04518410	Phase II and Phase III	Recruiting	8797	National Institute of Allergy and Infectious Diseases (NIAID)			A randomized study evaluating the efficacy of LY3819253 in COVID-19 patients who did not require hospital support.
29.	Anti-SARS-CoV-2 mAb	NCT04748588	Phase IV	Recruiting	648	University of Calgary	Canada	Randomized	A final trial aiming to evaluate the efficacy and safety of the antibody in the nosocomial COVID-19 patients in Canada.
30.	BI 767551	NCT04822701	Phase II and Phase III	Active but not recruiting	5	Boehringer Ingelheim	United States	Randomized	A randomized, placebo-controlled study reporting the tolerability, efficiency, and safety profile of the antibody in COVID-19 patients with mild to moderate symptoms.
31.	SAB- 185	NCT04469179	Phase I	Active but not recruiting	21	SAb Biotherap eutics, Inc.	United States	Randomized	A randomized study evaluating the efficacy of SAB-185 in COVID-19 patients who did not require hospital support.
32.	Bamlanivimab	NCT04796402	Phase IV	Active but not recruiting	576	Fraser Health	Canada	Randomized	A Phase IV study implicated for the emergency use of Bamlanivimab during the pandemic.

**Table 3:** Different neutralizing antibodies which are in the pre-clinical trial.

Sl. No.	nAb	International Nonpropreitary Name (INN)	Source	Type
1.	LY-CoV555	Bamlanivimab	Human B cells	mAb human IgG1
2.	JS016	Etesevimab + Bamlanivimab	Human B cells	mAb human, combination of 2 mAb
3.	LY-CoV016	Etesevimab + Bamlanivimab	Human B cells	mAb human, combination of 2 mAb
4.	LY3832479	Etesevimab + Bamlanivimab	Human B cells	mAb human, combination of 2 mAb
5.	REGN-COV2	Casirivimab + Imdevimab	Convalescent sources and immunization	mAb human
6.	TY027	-	-	mAb
7.	BRII-196	-	Human B cells	mAb human
8.	BRII-198	-	Human B cells	mAb human
9.	CT-P59	Regdanvimab	Human B cells	mAb human
10.	SCTA01	-	-	mAb humanized
11.	SAB- 185	-	Immunization	Polyclonal recombinant human Ab
12.	MW33	-	-	mAb human
13.	AZD7442	Tixagevimab + Cilgavimab	Human B cells	mAb human
14.	VIR-7831	Sotrovimab	Human B cells	mAb human
15.	DXP-593	-	Human B cells	mAb
16.	Anti-SARS-CoV-2 mAb	-	-	mAb, chicken IgY
17.	ABBV-47D11	-	Immunization	mAb human IgG1
18.	DXP604	-	Human B cells	mAb
19.	COVI-AMG (STI-2020)	-	In vitro libraries	mAb human
20.	C144-LS and C-135-LS	-	-	Mixture of 2 mAb
21.	ADG20	-	Human B cells	mAb human
22.	COR-101	-	In vitro libraries and human B cells	mAb human

**Table 4:** Emerging variants of SARS-CoV-2 and nAb, which are in the pre-clinical and clinical stage.

Sl. No.	Name of the Variant	Effective nAb against the SARS-CoV-2 Variants	Reference
1.	B.1.1.7 (Alpha)	CD147 (Meplazumab), COVOX-222, COVOX-253, A23-58.1, MG1141A, Sotrovimab, Casirivimab + Imdevimab, Bamlanivimab + Etesevimab, Tixagevimab + Cilgavimeb	[59,60,61]
2.	B.1.351 (Beta)	CD147 (Meplazumab), MG1141A, Casirivimab + Imdevimab, Sotrovimab, Tixagevimab + Cilgavimeb	[59,60,62]
3.	P.1 (Gamma)	CD147 (Meplazumab), COVOX-222, COVOX-253, A23-58.1, Sotrovimab, Casirivimab + Imdevimab, MG1141A, Tixagevimab + Cilgavimeb	[59,60,62]
4.	B.1.617.2 (Delta)	CD147 (Meplazumab), A23-58.1, Sotrovimab, Casirivimab + Imdevimab, Bamlanivimab + Etesevimab, Tixagevimab + Cilgavimeb	[60,62,67]
5.	B.1.1.529 (Omicron)	Sotrovimab, Paxlovid, molnupiravir	[73,74]

## Data Availability

Not applicable.

## Abbreviations

nAb	Neutralizing Antibody
mAb	Monoclonal Antibody
RBD	Receptor binding domain
ACE2	Angiotensin-converting enzyme 2
S-protein	Spike glycoprotein
NTD	N-terminal domain
CPT	Convalescent plasma therapy
ADE	Antibody-dependent enhancement
HCAbs	Heavy chain antibodies
IL-6	Interleukin 6
CDRH	Complementarity-determining regions of heavy-chain
FDA	Food & Drug Administration
IgA	Immunoglobulin A
MERS-CoV	Middle East respiratory syndrome coronavirus
VH	Variable domain heavy chain
VOI	Variant of Interest
VOC	Variant of Concern
